# Qualities of Older Adults’ Family and Friendship Relationships and Their Association with Life Satisfaction

**DOI:** 10.3390/geriatrics9020049

**Published:** 2024-04-10

**Authors:** Elias Mpofu, Rong-Fang Zhan, Cheng Yin, Kaye Brock

**Affiliations:** 1 Department of Rehabilitation and Health Services, University of North Texas, 1155 Union Circle #311456, Denton, TX 76203, USA; rongfang.zhan@unt.edu (R.-F.Z.); chengyinunt@gmail.com (C.Y.); kaye.brock@unt.edu (K.B.); 2 School of Health Sciences, University of Sydney, Camperdown, Sydney, NSW 2050, Australia; 3 Educational Psychology and Inclusive Education, University of Johannesburg, Auckland Park, Johannesburg 2006, South Africa; 4 School of Medical Sciences, University of Sydney, Sydney, NSW 2050, Australia

**Keywords:** family relationship quality, friendship quality, interaction, life satisfaction, older adults

## Abstract

While family and friendship relationship qualities are associated with life satisfaction, evidence on how these types of relationships interact to contribute to older adults’ life satisfaction is sparse. This study examined how family and friendship relationship qualities may be supportive of (compensatory) or conflict with (competing) older adults’ life satisfaction. We adopted a cross-sectional design to analyze data from the Health and Retirement Study (n = 1178, females = 54.8%, mean age = 67.9 years, SD = 9.3 years) to examine compensatory (as in social support) and competing (as in social strain) qualities of family and friendship social relationships and their association with life satisfaction in older adults. For greater explanatory power, we also controlled for life satisfaction by sociodemographic variables of age, gender, education, self-reported general health, physical health and activity, depression, and personality traits. Our findings indicate that the spouse/partner support relationship contributes to older adults’ life satisfaction overall and is associated with greater social support and less social strain. Friendship support is associated with improved life satisfaction for older adults reporting spouse/partner strain. Relationship support for the life satisfaction of older adults should consider their need for social support from their social network while minimizing the risk of social strain from adversarial relationships in life situations.

## 1. Statement of Relevance

This study examines relationship types for improving older adults’ life satisfaction through social support and risk due to adversarial or conflict-ridden relationships that could strain on older adults’ life satisfaction. This study addresses the following:

1. Interacting types of social support and social strain by types of relationships;
2. Unraveling the most significant relationships and controlling for demographics and self-rated health statuses;
3. Revealing the importance of family, child, non-child relative, and friend relationships to the overall life satisfaction of older adults;
4. Providing preliminary evidence to inform relationship support interventions responsive to older adults’ life situations to improve life satisfaction.

### 1.1. Older Adults’ Friendship Relationship Qualities: Their Association with Life Satisfaction 

As people age, they seek to optimize either their life satisfaction or overall subjective wellbeing [1]. However, in doing so, they may find that some of their relationships are supportive, adding to their life satisfaction [2,3], while others may subtract from their life satisfaction due to the social strain they experience [4,5]. The quality of relationships with spouses/partners, children, non-child relatives, and friends is important to overall life satisfaction among older adults [6,7,8,9,10] and for their ability to manage life strain [2]. Indeed, the aging and wellbeing paradox is the fact that in adults, older age is associated with higher subjective wellbeing or life satisfaction than younger age [11,12]. It appears that life satisfaction is relatively stable at older ages [13]. However, how older adults perceive their relationships, as complementary to or detracting from life situations, is not well understood. Therefore, we aimed to explore evidence of how older adults may perceive their relationship qualities to be compensatory of or subtractive in their life; the findings of this study might be helpful in the design of interventions for improving the life satisfaction of older adults.

### 1.2. Family and Friendship Relationships in Life Satisfaction

Evidence is mixed regarding the contributions of family, relations, and friends to life satisfaction in late life. For example, spousal/partner support was the most predictive of the mental health status of older adults, while the contributions of the other relationship types were less differentiated [14,15,16,17]. Tomini et al. [18] reported higher levels of life satisfaction among older adults with a larger network of close relatives than those with a larger number of friends. In addition, Pinquart and Sörensen [9] found that the parent–child relationship quality was more closely associated with the life satisfaction of older adults than the quality of friendship relationships. Arguably, these findings suggest that family, child, and non-child relative relationships are complementary to life satisfaction in older adults, though the same is not so true for friendship relationships, which could be competing with family relationships. In contrast, O’Connor [19] reported that friendship relationship qualities contributed more to the life satisfaction of older adults than parent–child relationship quality, suggesting a likely complementary effect of the friendship relationship. Similarly, Huxhold et al. [20] found that friendship relationships contributed more to the life satisfaction of older adults and family member relationships did not [21]. The gender effect on social relationship quality at an older age remains speculative. As far back as two decades ago, Umberson et al. [22] reported to “find little evidence for the assertion that men and women react to strained relationships in gender” (p. 43). A more recent study by Waite and Das [23] concluded that the “findings yield a mixed picture of gender-differentiated vulnerabilities balanced by proactive adaptation and maintenance of social and dyadic assets” (p. 87). Importantly, there is a lack of consensus regarding whether spouse/partner, child, relative, and friend relationships are complementary to (as in support) or compete with (as in strain) life satisfaction in older adults. 

Theoretical foundations: The hierarchical compensatory model [24] regards a relationship as supportive if people are involved to mutually augment their overall social wellbeing. If that were the case, then spouse/partner, child, and relative relationships would be complemental in light of their effects on the life satisfaction of older adults [15,25]. Conceivably, a friendship relationship may also add to the relationship support gains of the family relationship, rather than competing those relationships. However, as previously noted, relationship type effects within a family and by friendship type may not necessarily be complementary in their roles in the life satisfaction of older adults [14,15,20] and may be competing, which may be associated with social distress or strain.

Socioemotional selectivity theory [26] proposes that older adults with competing relationships may seek to regulate their relationships by investing in relationship types they perceive to optimize their life satisfaction rather than in all relationship types. For instance, older adults may be drawn toward emotionally intimate relationships (e.g., spouse/partner) rather than relationships with others (e.g., children, non-child relatives, own parents, friends) which they consider less important [25,27]. This preference bias may be explained by the fact that for older adults, the spouse/partner tends to be physically and psychologically and more important to their satisfaction than children, relatives, and friends [28,29,30]. Nonetheless, older adults may seek to select or prioritize relationships that minimize social strain for a greater sense of life coherence [12]. Conceivably, the family and parent–child relationship would be closer to the older adult’s life situation than the relative and friend relationships [26], enhancing their immediate psychological resources for wellbeing [12]. 

However, partner loss and declining health are the main causes of unhappiness in older age [11]. Nonetheless, the relative and friend relationships may be important to older adults’ life situations, mitigating social strain from the family and parent–child relationships [31].

### 1.3. Significance of Personal Factors, Health and Function, and Social Interaction Types

As previously noted, life satisfaction appears to increase with older age (i.e., the aging–wellbeing paradox), controlling for sociodemographics of race/ethnicity, socio-economic class, health, and function [32]. This is not to discount the fact that older adults with chronic health conditions need dependable resources to manage their social worlds, thus minimizing their vulnerabilities due to the aging processes [33]. Physical limitations to self-managing activities of daily living, socializing with friends, family, and routines that bring joy and comfort may reduce the risk of depression and improve subjective wellbeing [34]. Having an extroverted personality was associated with higher levels of social engagement, while openness tended to decrease [35,36]. However, there is also evidence to suggest that extraversion, neuroticism and openness tend to decrease as people increase in age [37], while characteristics including agreeableness and conscientiousness increased, the association of which with life satisfaction is less known [38].

The present study. We thus sought to explore how older adults’ life satisfaction may be associated with social support and/or strain from spousal/partner child, relative, and friends relationships. Research “needs to consider both positive and negative relationship features from diverse sources separately and in combination to disentangle their relative effects and their additive or compensatory potential” [15]. Our specific research questions were as follows:

1. How are older adults’ social demographics and personal and social interaction factors associated with their life satisfaction?
2. How are social support and social strain in spousal/partner, child, relative, and friendship relationships associated with life satisfaction in older adults?

We proposed and tested the following hypotheses:

**Hypothesis** **1.** *Increased age, health and functioning, personality traits and spousal/partner support are significantly associated with life satisfaction in older adults.*

**Hypothesis** **2.** *Spouse/partner support is associated with life satisfaction in older adults significantly more than child, relative, and friendship support relationships.*

**Hypothesis** **3.** *Within types of relationships (spousal/partner, child, friends, and relative), a lower social strain is associated with the life satisfaction of older adults.*

**Hypothesis** **4.** *The interaction factor between friends’ social support and spousal/partner social strain (distress) is associated with life satisfaction in older adults, with high levels of spouse/partner strain and high levels of friend social support and low levels of friends support and little spouse/partner social strain.*

The national and international significance of this study lies in that little is known globally about the relationship qualities and life satisfaction in older adulthood. This is despite the fact that age-friendly communities place an emphasis on social participation and inclusion across the lifespan [39,40,41], and evidence is needed from the international community for insights into alternative relationships and ways of social connectedness with and by the world’s older adult population [41].

## 2. Methods

### Sources of Data 

In this exploratory cross-sectional design study [42], we utilized secondary data from the 2014 Wave of the Health and Retirement Study (HRS) [43] of older Americans 50 years old and older. Exploratory cross-sectional design studies are ideal for mapping evidence on less well-established relationships or those with limited emerging evidence, as was the case with our study to unravel the potentially complemental or competing relationship types and their associations with life satisfaction in older adults. While there “seems to be a universal condemnation of the cross-sectional design and at the same time acceptance of the superiority of the longitudinal design in allowing conclusions about temporal precedence and even causality”, what is “often overlooked is that the cross-sectional design can tell us much that is of value and that the longitudinal design is not necessarily superior in providing evidence for causation” ([42], p. 125).

The University of Michigan collects HRS data, funded by the National Institute on Aging and the Social Security Administration. The HRS provides information about respondents and their spouses/partners such as health, disability, work status and history, and economic status, as well as psychosocial factors, job, pension, and health insurance characteristics. For this study, we selected 1178 of 18,747 cases (females = 54.8%; mean age = 67.9 years; SD = 9.3 years) with complete or non-missing data on life satisfaction, social interaction, activities of daily living (ADLs), depression, subjective social status (SSS), personality traits, and education variables, minimizing inference ambiguities that come with data imputation methods [44,45]. For this exploratory cross-sectional study, we analyzed the 1178 cases, avoiding the further loss of cases with a longitudinal multi-wave analysis. The higher number of covariates for these analyses were guided by previous systematic review studies on correlates of the social wellbeing of older adults [4,46]. 

Table 1 presents the demographic and general health characteristics of the 1178 respondents. The sample was primarily middle-aged, female, and in good health. Most of the respondents had completed their high school education and had a higher level of life satisfaction.

## 3. Measures

The HRS made use of a variety of established measures. We briefly describe these next. 

Life satisfaction: Participant older adults completed the validated Satisfaction with Life Scale, which comprises five items: (a) “In most ways my life is close to ideal”; (b) “The conditions of my life are excellent”; (c) “I am satisfied with my life”; (d) “So far, I have gotten the important things I want in life”; and (e) “If I could live my life again, I would change almost nothing”. Each item is rated on a 7-point scale ranging from 1 (*strongly disagree*) to 7 (*strongly agree*). We calculated average scores across these five items to create an index of life satisfaction, with a higher score indicating a higher level of life satisfaction. The Cronbach’s alpha for scores from the SWL scale in the present study was 0.89.

Social interaction qualities: We selected indicators of positive social interactions as measures of social support and indicators of negative social interactions as measures of social strain across relationship domains separately. These social interaction measures were as follows.

*Social support*: Participants self-rated their positive social interactions in reference to four relationship domains: spouse/partner, children, other relatives, and friends. Within each relationship domain, the participants responded to the following three items on a 4-point Likert scale (1 = *a lot*; 4 = *not at all*): (a) “How much do they really understand the way you feel about things?”; (b) “How much can you rely on them if you have a serious problem?”; and (c) “How much can you open up to them if you need to talk about your worries?”. The responses for positive relationships were reverse-coded so that higher scores indicated higher levels of social support in the relationship quality. All scores regarding positive social interaction were averaged to create a social support quality score for each domain (Cronbach’s alpha range = 0.81–0.86).

*Social strain*: The participants also self-rated their negative social interactions in the same four social relationship domains. Each domain included the following four items on a 4-point Likert scale (1 = *a lot*; 4 = *not at all*): (a) “How often do they make too many demands on you?”; (b) “How much do they criticize you?”; (c) “How much do they let you down when you are counting on them?”; and (d) “How much do they get on your nerves?”. Higher scores meant lower levels of social strain. We averaged the negative social interaction scores across relationship domains to create a social strain quality score for each domain (Cronbach’s α range = 0.78–0.81). 

Covariates: In our selection of covariates, we were guided by previous studies [15,25,32] and the research evidence from a systematic review [4,46]. We included as covariates sociodemographic variables of age (years) and education (number of school years) (as continuous variables) and gender (coded as 0 for male and 1 for female), and measures of general health, physical health and activity, depression and personality traits. On the HRS, these variables were measured as follows.

*General health* was measured on a five-point scale (1 = *excellent*, 2 = *very good*, 3 = *good*, 4 = *fair*, and 5 = *poor*) and then dichotomized into excellent/very good/good (recoded as 1) versus fair/poor (recoded as 0); *subjective social status* was ranked from 10 (highest: the most money, the highest level of education, and the best jobs) to 1 (the lowest rating: the bottom were the poorest, with the least education and the worst jobs). *Physical health* was self-rated as having difficulty performing *activities of daily living* ((ADLs) dressing, walking across a room, taking a bath or shower, eating, and getting in and out of bed: 1 = *yes* and 0 = *no*), and *instrumental activities of daily living* ((IADLs) difficulty preparing meals, shopping for groceries, making phone calls, managing money, and taking medications: 1 = *yes* and 0 = *no*). Both of the above measures yielded two scores ranging from 0 (*the most independent*) to 5 (*the most dependent*) (Cronbach’s alpha = 0.70 for ADLs; Cronbach’s alpha = 0.66 for IADLs). We log-transformed these two variables to conform to normality. Log-transformed scores are robust to violations of the normality of the distribution of scores and provide a fail-safe mechanism for the analysis that exceeds alternative methods [47]. 

*Depression* was measured on the 8-item Center for Epidemiologic Studies Depression Scale (CES-D, including a depressed mood, slowness in activity, sleep disturbance, happiness, loneliness, life enjoyment, sadness, and too much effort in life as follows: 0 = *no depressive symptomatology;* 8 = *severe depressive symptomatology*) (Cronbach’s alpha = 0.80). 

*Physical activity* was assessed using three questions about the frequency of vigorous-, moderate- and mild- intensity physical activity as follows: 1 = *hardly ever or never* to 5 = *every day*, and the higher scores indicate more frequent physical activity participation). The *Personality Scales* included extraversion, openness to experience, neuroticism, agreeableness, and conscientiousness, and answer choices were presented on a four-point scale ranging from 1 (*a lot*) to 4 (*not at all*). We added up all the scores for each personality trait and then averaged the scores (Cronbach’s alpha.73 to 0.80). 

### 3.1. Procedure

The HRS data held by the University of Michigan are publicly available and require no Institutional Review Board approval to use them. 

### 3.2. Analysis Strategy

We utilized the Statistical Package for the Social Sciences (SPSS) version 25.0 to perform multiple linear regression tests. Prior to the regression analysis, we used several statistical tests (e.g., the variance inflation factor, Durbin–Watson statistic, and Cook’s Distance) to ensure valid data assumptions [48]. For the regression modeling, we used a simultaneous regression model in order to explore the comparative contribution of spouse/partner, child, relative, and friend relationship qualities to the life satisfaction of older adults, controlling for age, gender, education, subjective social status, health status, ADL, IADL, depression, physical activity, and personality traits. For the hypothesized interaction effect, we computed mean-centered main effects using 12 sets of hierarchical regression analyses.

Although this may seem like an inordinately large number of effects for a single study, we were guided by the literature on the need to test for “domain-specific and crossover effects, so that both positive and negative exchanges are related to both positive and negative indicators of mental health” ([15], p. 661). Moreover, social support and social strain interaction exchanges tend to associate with each other in their influence on wellbeing indicators and need to be assessed separately and also jointly [29]. We applied the Dunn–Bonferroni procedure to control for Type 1 error inflation within clusters of relationships for the prediction of life satisfaction. With the highest cluster of factors at a total of nine, our 95% confidence for the test statistics applying the Dunn–Bonferroni procedure was 0.005 (or 0.05/9 = 0.005). 

## 4. Results

### 4.1. Sociodemographic, Health and Function, and Life Satisfaction 

Older adults in this sample who reported higher levels of life satisfaction were of increasing age (β = 0.09, *p* = 0.001), a lower education level (β = −0.09, *p* = 0.001), and had self-perceived higher social status (β = 0.17, *p* = 0.000). They had low ADL limitations (β = −0.12, *p* = 0.000), no low depressive symptoms (β = −0.13, *p* = 0.000), and extroverted tendencies (β = 0.10, *p* = 0.006) (see Table 2). These findings are in partial support of Hypothesis 1 (increased age, health and function, personality traits, and spousal/partner support are significantly associated with life satisfaction in older adults). Of note is the fact that the gender effect was not statistically significant (β = 0.04, *p* > 0.05), and we therefore did not include that variable in subsequent interaction analyses.

### 4.2. Relationships Types and Life Satisfaction 

Table 2 presents the results of the simultaneous regression model. The entire set of predictor variables (Spousal/Partner, Child, Relative, and Friend relationships) accounted for a significant amount of variance in life satisfaction (*R* = 0.53, *R*^2^ = 0.28, F(23, 1154) = 19.84, *p* = 0.000). Standardized partial regression coefficients revealed that spouse/partner support was associated with higher levels of life satisfaction in older adults (β = 0.14, *p* = 0.000), Spouse/partner strain and child support were less associated with life satisfaction in older adults (β = 0.08, *p* = 0.014 and β = 0.06, *p* = 0.048 respectively). These findings are in support of Hypothesis 2 (spouse/partner support is associated with life satisfaction in older adults significantly more than child, relative, and friend support). The results of a T-test comparison of the beta weights for spouse/partner and child relationship quality variables were not statistically significant. 

### 4.3. Combined Effects of Relationship Quality on Life Satisfaction 

**Figure 1:**
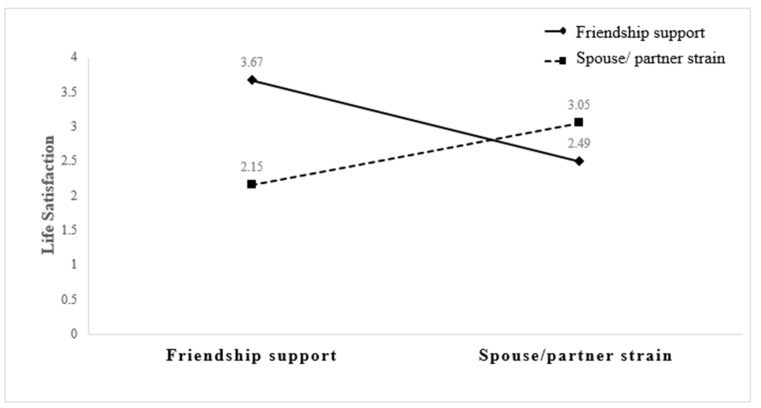
Relative support and friendship strain interaction on life satisfaction.

**Figure 2:**
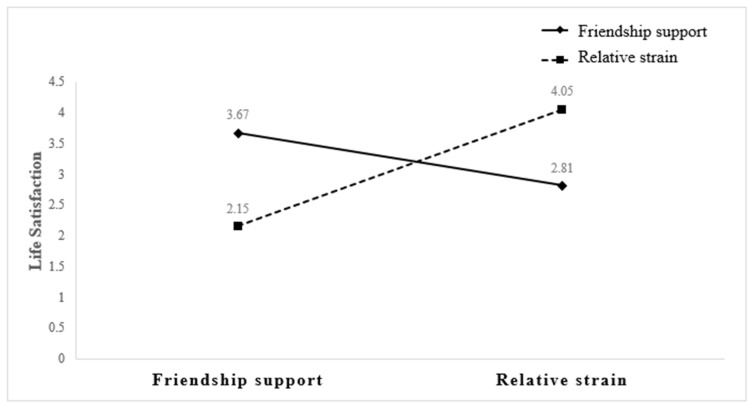
Friendship support and spousal strain interaction on life satisfaction.

**Figure 3:**
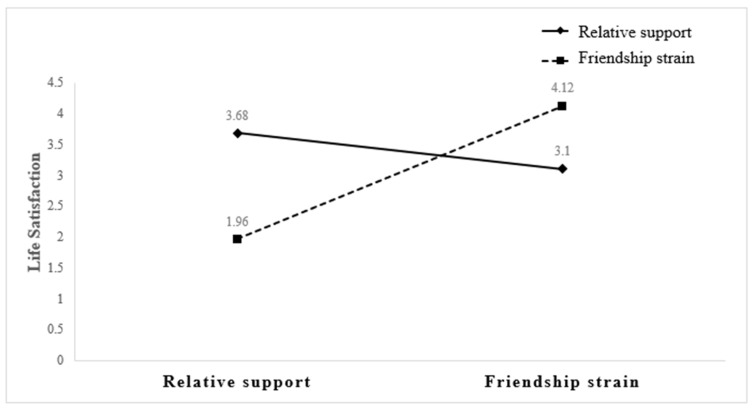
Friendship support and relative strain interaction on life satisfaction.

Table 3 presents the results from the interaction factor analysis controlling for main effects. The interaction between greater relative support and lower friendship strain was associated with higher life satisfaction in older adults (β = 0.07, *p* = 0.014) (see also Figure 1). Similarly, greater friendship support and less spouse/partner strain was associated with life satisfaction in adults of an older age (β = 0.07, *p <* 0.01) (see Figure 2), as was the interaction between higher friendship support and lower relative strain (β = 0.06, *p =* 0.039) (see Figure 3). Overall, adults of an older age with high levels of spouse/partner social strain and high levels of friendship support have greater life satisfaction than older adults with low levels of spousal/partner social strain and low levels of friend support.

This result is in support of Hypothesis 3 (the interaction factor between friendship support and spousal/partner strain is associated with life satisfaction in older adults in circumstances of high family strain and high friend support and low friend support and low spouse/partner strain). 

## 5. Discussion

The findings of our investigation indicate higher levels of life satisfaction among adults of an older age, which is consistent with the aging and wellbeing paradox literature [11,12,32]. The aging and wellbeing paradox literature reports that contrary to commonly held opinions, adults of an older age were happier with their lives overall, predicated on their declining physical functioning. This life satisfaction at an older age was associated with in lower depression symptoms, extroverted tendencies, and fewer limitations on their activities of daily living [34,35,49]. These findings suggest that life satisfaction at an older age is explained by subjective wellbeing and personal resource capabilities for overall wellbeing [49]. In addition, contrary to commonly assumed differences by gender, these results were not found to be gendered in our present study. 

The results of this study suggest that the quality of a relationship with a spouse/partner significantly contributed to life satisfaction among older adults, perhaps more than relationships with children, non-child relatives, and friends [9,16,17,28]. This could be explained by the fact that spouse/partner relationships become more salient with aging when older adults risk losing other social relationships (e.g., relationships with non-child relatives, friends and neighbors) because of death and relocation [15,25,29]. Other than declining health, loss of partner is the main cause of lack of life satisfaction in older age [11]. 

The findings of our study are consistent with the hierarchical compensatory model, which proposes that an inner circle of immediate family relationships contributes to the life satisfaction of older adults more than non-child relatives and friends who are the outer relationship circle do [18]. In addition, our findings are also consistent with socioemotional selectivity theory [26] to the effect that older adults invest more in their spouses/partners for life satisfaction than other relationships [18,25,29]. It has been suggested that over time, older adults value the spouse/partner relationship above all other relationship qualities because of their emotional connection over time [50]. Other authors have indicated that older adults with spouse/partner social strain may elect relative or friends for an alternative compensatory relationship for their wellbeing [51].

The present study found that a positive friendship relationship might offset the ill effects of social strains from a spouse/partner or relative relationship on the life satisfaction of older adults. Birditt et al. [27] also propose that friendship may help with strain from relationships with children. Other authors have also suggested modifying effects [14,15]. Our findings may be explained by the fact that friendship is characterized by affection, trust, commitment, respect, and reciprocity in old age [52]. Conceivably, the friendship relationship, as it is typically voluntary, is of a lower demand to sustain or withdraw from if needed compared to the obligatory spousal and child relationships. Older adults may perceive more control in their participation in a friendship relationship, and they may have mixed feelings about their spousal/partner, child, and relative relationships [53]. Positive friendship relationships serve to reduce loneliness and provide emotional and instrumental support regarding social strain from a spouse/partner or children [28]. 

### 5.1. Implications for Gerontologic Research and Practice

We draw several research implications from these findings, including a need for studies to further clarify the life satisfaction needs of older adults by their relationship preferences and priorities. Understanding the relationship qualities required for a satisfying life in older adulthood would inform the design of support interventions tailored to the needs of older adults, their life situations, and sociodemographic diversities. Findings from this study help benchmark stability and change in the quality of adult relationships with respect to living arrangements, health function, and support resources (controlling for sociodemographics). Moreover, the findings from this study will provide a basis for older adult life satisfaction product development, dissemination, and utilization studies. Geriatric health practitioners may find these data helpful in the design of life satisfaction interventions for older adults by the older adults’ relationship dispositions, consistent with age-friendly care policies. 

### 5.2. Limitations of this Study and Suggestions for Further Research

This study has some limitations. First, we analyzed cross-sectional data from 1178 cases with no missing data for the advantage of data integrity and explanatory power. However, the constraints on the data may have biased our findings in unknown ways. Studies of a cross-sectional design have limitations in looking at prospective effects. Although life satisfaction and relationships at an older age are known to be relatively stable [13], it should nonetheless be suggested that future longitudinal data analyses should be conducted on the significant relationship type effects to clarify on their stability over time.

Second, the HRS data are self-reported and thus subject to social desirability bias. Third, the HRS data utilized a global measure of life satisfaction, and this may mask differences in relationship quality influences across life domains. We suggest that future analyses should utilize a multi-dimensional measure of life satisfaction to explore the relationships considered in this study with more precision. Moreover, future studies should also include HSR measures of relationships with neighbors as well as relationships with a spouse/partner, children, non-child relatives, and friends. Neighbors are in close physical proximity whereas children, other relatives, and friends often live far away. The proximity effect (e.g., physical and psychological proximity) may contribute to an increase in interpersonal connections [54,55]. Third, although we did not observe a significant gender effect in this data analysis, we suggest that studies should routinely include a gender identity variable in their data collection and analysis. 

## 6. Conclusions

The results of this study indicate that spouse/partner and parent–child relationship qualities significantly predict the life satisfaction of older adults more than relative and friendship relationships. These relationship preferences would be expected in view of the higher closeness of family as compared to relative or friendship relationships at older adult ages. Many adults of an older age want to self-manage their everyday living with a social community of family, relatives, and friends. Self-managing their own social wellbeing ultimately rests in part on life situations and personal preferences for a satisfying life. In this study, we observed cross-relationship domain-buffering effects on life satisfaction in which positive relative and friendship relationships likely contribute to the life satisfaction of older adults with spouse/partner and/or child support relationship strain. Thus, while these results suggest familial ties to largely explain the life satisfaction of adults of an older age, other relationship types matter to understanding quality of life in older adulthood. 

## Figures and Tables

**Table 1:** Descriptive characteristics of variables (n = 1178).

Variables	Total (Percentage/%)	Mean (Standard Deviation)
Life satisfaction		4.93 (1.52)
Social interaction quality		
Spouse/partner support		3.41 (0.67)
Spouse/partner strain		3.01 (0.69)
Child support		3.22 (0.73)
Child strain		3.27 (0.64)
Relative support		2.82 (0.86)
Relative strain		3.43 (0.62)
Friend support		2.91 (0.76)
Friend strain		3.61 (0.51)
Age		67.9(9.3)
50–64	435 (36.9%)	
65–74	401 (34.0%)	
75–84	288 (24.4%)	
85+	54 (4.6%)	
Gender		
Female	645 (54.8%)	
Male	533 (45.2%)	
Education		12.63 (2.88)
Subjective social status		6.32 (1.76)
Self-reported general health status		
Good	718 (61.0)	
Poor	460 (39.0)	
Physical health		
Activities of daily living		0.45 (0.95)
Instrumental activities of daily living		0.30 (0.77)
Depressive symptoms		1.73 (2.06)
Physical activity		
Vigorous activity		3.26 (1.30)
Moderate activity		2.66 (1.61)
Mild activity		2.24 (1.64)
Personality traits		
Extraversion		3.13 (0.58)
Openness to experience		2.85 (0.57)
Neuroticism		2.08 (0.63)
Agreeableness		3.50 (0.50)

**Table 2:** Multiple regression analysis for prediction of life satisfaction (n = 1178).

Variables	B	SE B	Standardized β
Age	0.16	0.05	0.09 **
Gender	0.12	0.09	0.04
Education	−0.05	0.02	−0.09 **
Subjective social status	0.15	0.02	0.17 **
Self-reported general health status	0.20	0.09	0.07 ^†^
Physical health			
Activities of daily living	−0.91	0.24	−0.12 **
Instrumental activities of daily living	−0.09	0.27	−0.01
Depressive symptoms	−0.63	0.16	−0.13 **
Physical activity			
Vigorous activity	0.02	0.04	0.02
Moderate activity	0.03	0.03	0.02
Mild activity	0.02	0.04	0.01
Personality traits			
Extraversion	0.26	0.09	0.10 *
Openness to experience	−0.09	0.09	−0.03
Neuroticism	−0.12	0.07	−0.05
Agreeableness	0.15	0.10	0.05
Social interaction quality			
Spouse/partner support	0.32	0.07	0.14 **
Spouse/partner strain	0.18	0.07	0.08 ^†^
Child support	0.13	0.07	0.06 ^†^
Child strain	−0.06	0.08	−0.03
Relative support	0.01	0.05	0.01
Relative strain	0.07	0.08	0.03
Friendship support	0.05	0.06	0.03
Friendship strain	−0.14	0.09	−0.05

Note. ^†^ *p* < 0.05, * *p* ≤ 0.01, ** *p* ≤ 0.001. Explained variable by the model = R^2^ = 0.28.

**Table 3:** Hierarchical regression results for prediction of life satisfaction with emotional loading (n = 1178).

Relationship	B	SE	Beta	*R* ^2^	Δ*R*^2^	ΔF
Spouse/Partner						
Step 1						
Spouse/partner support	0.62	0.06	0.28 **	0.10	0.10	67.89 **
Child strain	0.29	0.07	0.12 **			
Step 2						
Spouse/partner support * Child strain	0.10	0.09	.00	0.10	0.00	0.01
Step 1						
Spouse/partner support	0.62	0.06	0.27 **	0.11	0.11	69.39 **
Relative strain	0.32	0.07	0.13 **			
Step 2						
Spouse/partner support * Relative strain	0.10	0.09	0.03	0.11	0.00	1.31
Step 1						
Spouse/partner support	0.66	0.06	0.29 **	0.10	0.10	62.76 **
Friendship strain	0.26	0.08	0.09 *			
Step 2						
Spouse/partner support * Friendship strain	0.14	0.12	0.03	0.10	0.00	1.29
**Child**						
Step 1						
Child support	0.39	0.06	0.19 **	0.11	0.11	71.54 **
Partner strain	0.52	0.06	0.23 **			
Step 2						
Child support * Spouse/partner strain	−0.01	0.08	0.00	0.11	0.00	0.02
Step 1						
Child support	0.42	0.06	0.20 **	0.07	0.07	46.78 **
Relative strain	0.34	0.07	0.14 **			
Step 2						
Child support * Relative strain	0.03	0.09	0.01	0.07	0.00	0.15
Step 1						
Child support	0.47	0.06	0.23 **	0.06	0.06	39.85 **
Friendship strain	0.26	0.09	0.09 *			
Step 2						
Child support * Friendship strain	0.07	0.10	0.02	0.06	0.00	0.49
**Relative**						
Step 1						
Relative support	0.19	0.05	0.11 **	0.09	0.09	55.29 **
Spouse/partner strain	0.58	0.06	0.26 **			
Step 2						
Relative support * Spouse/partner strain	−0.10	0.07	−0.04	0.09	0.00	1.80
Step 1						
Relative support	0.19	0.05	0.11 **	0.04	0.04	25.57 **
Child strain	0.38	0.07	0.16 **			
Step 2						
Relative supsrain	0.08	0.08	0.03	0.04	0.00	1.13
Step 1						
Relative support	0.22	0.05	0.13 **	0.03	0.03	18.18 **
Friendship strain	0.33	0.09	0.11 **			
Step 2						
Relative support * Friendship strain	0.26	0.10	0.07 ^†^	0.04	0.01	6.06 ^†^
**Friendship**						
Step 1						
Friendship support	0.28	0.06	0.14 **	0.10	0.10	61.38 **
Spouse/partner strain	0.58	0.06	0.26 **			
Step 2						
Friendship support*spouse/partner strain	0.20	0.08	0.07 *	0.10	0.01	6.95 *
Step 1						
Friend support	0.29	0.06	0.15 **	0.05	0.05	32.07 **
Child strain	0.39	0.07	0.16 **			
Step 2						
Friendship support * child strain	0.14	0.08	0.05	0.05	0.00	2.86
Step 1						
Friendship support	0.29	0.06	0.15 **	0.06	0.06	34.70 **
Relative strain	0.42	0.07	0.17 **			
Step 2						
Friendship support * Relative strain	0.17	0.08	0.06 ^†^	0.06	0.00	4.25 ^†^

Note. ^†^ *p* < 0.05, * *p* < 0.01, ** *p* < 0.001.

## Data Availability

This manuscript based on a secondary dataset of reports containing de-identified data and does not contain any individual person’s data in any form or case reports requiring consent for publication. Data access can be obtained from the Inter-University Consortium for Political and Social Research for the Health and Retirement Study upon reasonable request.
